# The association between leisure-time physical activity, low HDL-cholesterol and mortality in a pooled analysis of nine population-based cohorts

**DOI:** 10.1007/s10654-017-0280-9

**Published:** 2017-06-30

**Authors:** Gary O’Donovan, David Stensel, Mark Hamer, Emmanuel Stamatakis

**Affiliations:** 1 School of Sport, Exercise and Health Sciences, National Centre for Sport & Exercise Medicine–East Midlands, Loughborough University, Loughborough, LE11 3TU UK; 2 Department of Epidemiology and Public Health, University College London, London, WC1E 6BT UK; 3 Charles Perkins Centre, Prevention Research Collaboration, School of Public Health, University of Sydney, Sydney, Australia

**Keywords:** Physical activity, Cholesterol, Mortality, Biological interaction

## Abstract

The objective of this study was to investigate associations between leisure-time physical activity, low high-density lipoprotein cholesterol (HDL-C) and mortality. Self-reported leisure-time physical activity, HDL-C concentration, and mortality were assessed in 37,059 adults in Health Survey for England and Scottish Health Survey. Meeting physical activity guidelines was defined as ≥150 min wk^−1^ of moderate-intensity activity, ≥75 min wk^−1^ of vigorous-intensity activity, or equivalent combinations. Low HDL-C was defined as <1.03 mmol L^−1^. Cox proportional hazard models were adjusted for age, sex, smoking, total cholesterol, systolic blood pressure, body mass index, longstanding illness, and socioeconomic status. There were 2250 deaths during 326,016 person-years of follow-up. Compared with those who met physical activity guidelines and whose HDL-C was normal (reference group), all-cause mortality risk was not elevated in those who met physical activity guidelines and whose HDL-C concentration was low (hazard ratio: 1.07; 95% confidence interval: 0.75, 1.53). Compared with the reference group, all-cause mortality risk was elevated in those who did not meet physical activity guidelines and whose HDL-C was normal (1.37; 1.16, 1.61), and in those who did not meet physical activity guidelines and whose HDL-C was low (1.65; 1.37, 1.98). Cardiovascular disease mortality hazard ratios were similar, although confidence intervals were wider. There was no statistically significant evidence of biological interaction between physical inactivity and low HDL-C. This novel study supports the notion that leisure-time physical activity be recommended in those with low HDL-C concentration who may be resistant to the HDL-raising effect of exercise training.

**Electronic supplementary material:**

The online version of this article (doi:10.1007/s10654-017-0280-9) contains supplementary material, which is available to authorized users.

## Introduction

In the Third Report of the US National Cholesterol Education Program Expert Panel on Detection, Evaluation, and Treatment of High Blood Cholesterol in Adults (Adult Treatment Panel III [ATP III]), it stated that low high-density lipoprotein cholesterol (HDL-C) was an independent risk factor for coronary heart disease [1]. Physical activity and other therapeutic lifestyle changes were recommended to reduce cardiovascular disease (CVD) risk in those with low HDL-C in ATP III and in a subsequent report from the Coordinating Committee of the National Cholesterol Education Program [2]. Physical inactivity is regarded as a cause of low HDL-C levels [1] and exercise training tends to increase HDL-C [3]. Leisure-time physical activity is associated with reduced risks of mortality from CVD and all causes [4, 5]. Low HDL-C is associated with increased risks of mortality from CVD and all causes [6, 7]; However, the relationships between physical activity, low HDL-C and mortality are unclear. The benefits of HDL-raising drugs are ambiguous [8] and some people may be resistant to the HDL-raising effect of exercise [9–12]. The objective of this study was to investigate associations between leisure-time physical activity, low HDL-C and mortality in a pooled analysis of nine population-based cohorts in Britain.

## Methods

### Participants

The Health Survey for England (HSE) and the Scottish Health Survey (SHS) are household-based surveillance studies that are described in detail elsewhere [13, 14]. The present study included participants from surveys in 1995 (SHS only), 1998 (HSE and SHS), 1999 (HSE only), 2003 (HSE and SHS), 2004 (HSE only), 2006 (HSE only), and 2008 (HSE only). The same organization carried out the surveys using consistent methods [13, 14]. The HSE and SHS samples were selected using a multistage, stratified probability design to be representative of the target populations of the corresponding countries. Stratification was based on geographical areas and not on individual characteristics: postcode (zip code) sectors were selected at the first stage and household addresses selected at the second stage. Local research ethics committees approved all aspects of each survey and all participants gave written informed consent.

### Physical activity

Trained interviewers asked participants about physical activity. The questionnaires used to assess physical activity in the HSE and SHS surveys are described in detail elsewhere [15]. Briefly, the interviewer used the questionnaire to inquire about the following aspects of the respondent’s physical activity in the four weeks before the interview: frequency and duration of participation in domestic physical activity (light and heavy housework, gardening, and do-it-yourself tasks); frequency, duration and pace of walking (slow, average, brisk, or fast); and participation in sports and exercises using a prompt card showing 10 main groups, including cycling, swimming, running, football, rugby, tennis, and squash. Six open entries could also be recorded. For each sport and exercise, the respondent was asked to specify frequency, duration, and perceived intensity. The validity [16] and reliability [17] of the questionnaires have been reported. One metabolic equivalent (MET) was considered to represent resting energy expenditure and activities were quantified in terms of multiples of resting energy expenditure. A compendium of physical activities was used to assign MET values [18]. Light-intensity activities (such as “light” gardening activity and slow-paced walking) were assigned MET values of 1.5–2.9; moderate-intensity activities (such as brisk/fast walking and a subset of sports and exercises) were assigned MET values of 3.0–5.9; and, vigorous-intensity activities (such as swimming, jogging and running) were assigned MET values of ≥6.0 METs. Leisure-time activities and domestic activities are included in prevailing physical activity guidelines [19]; however, domestic activities were not included in the present analysis because of the reported lack of association between domestic activity and mortality [20, 21]. Meeting physical activity guidelines was defined as taking part in at least 150 min per week of moderate-intensity leisure-time physical activity, or at least 75 min per week of vigorous-intensity leisure-time physical activity, or any combination of moderate- and vigorous-intensity physical activity equivalent to at least 7.5 MET-h wk^−1^ [19]. A MET-hour is computed by multiplying the MET score of an activity by the time performed [18].

### Clinical data

Trained interviewers measured height and weight and asked about smoking habit and longstanding illness. Body mass index (BMI) was expressed as kilograms per meter squared. Longstanding illness was defined as any illness, disability or infirmity that had troubled the respondent over a period of time or was likely to affect them over a period of time. A longstanding illness was defined as limiting if the respondent said that it limited their activities in any way. Trained and qualified nurses asked about the use of CVD medication, including beta blockers, ACE inhibitors, diuretics, calcium blockers, and lipid lowering agent. The nurses also measured blood pressure and obtained a non-fasting venous blood sample. Blood pressure was measured three times after 5 min of seated rest. In this study, systolic blood pressure was computed as the average of the second and third readings. Blood samples were sent to the Biochemistry Department at the Royal Victoria Infirmary in Newcastle for the measurement of total cholesterol and HDL-C (640 analyser, Olympus Corporation, Tokyo, Japan). Detailed information on the methodology of the blood analysis, the internal quality control, and the external quality assessment for the laboratory have been described elsewhere [22]. The coefficient of variation of the assays was <4%. In keeping with previous work [7], the ATP III sex-specific definition was used to define low HDL-C: <1.03 mmol L^−1^ in men and <1.30 mmol L^−1^ in women [1].

### Socioeconomic status

Socioeconomic status was assessed using the four-group version of the Registrar General’s classification: professional and managerial occupations; skilled, non-manual occupations; skilled manual occupations; and, routine and manual occupations.

### Mortality follow-up

Participants were flagged by the British National Health Service Central Registry. For participants who survived, the data were censored up to the end of 2009 (Scottish Health Survey) or the first quarter of 2011 (Health Survey for England). Diagnoses for the primary cause of death were based on the *International Classification of Diseases*, Ninth (ICD-9) and Tenth (ICD-10) Revisions. Codes corresponding to CVD mortality were 390-459 for ICD-9 and I01-I99 for ICD-10.

### Statistical analysis

The Cox proportional hazards model was used to estimate the associations of physical activity and HDL-C on the risks of all-cause and CVD mortality. The proportional hazards assumption was examined by comparing the cumulative hazard plots grouped on exposure, although no appreciable violations were noted. For the present analyses, calendar time (months) was the timescale. Data from 371 participants who died during the first 24 months of follow-up were not included in the present analysis to address the issue of reverse causation, where participants with underlying disease are less likely to be physically active (there were 371 deaths from all causes, including 61 from CVD). Analyses were adjusted for age and sex (Model 1) and further adjusted for smoking, total cholesterol, systolic blood pressure, body mass index, longstanding illness, and socioeconomic status (Model 2). It has been argued that models should include variables that are thought to be important in the literature [23]. The literature suggests that the variables included in Model 1 and Model 2 are important chronic disease risk factors [1]. Three sensitivity analyses were performed. First, low HDL-C was defined as <1.03 mmol L^−1^ in both men and women [1] to investigate whether the use of a single definition influenced the associations between physical activity, HDL-C and mortality. Second, the associations between physical activity, HDL-C and mortality were investigated in the subsample with medication data to further address the issue of reverse causation. Third, the associations between physical activity, HDL-C and mortality were investigated in the subsample aged 40 years and older because it was thought that congenital abnormalities would be responsible for events in young individuals and lifestyle would be responsible for events in middle-aged and older adults [24]. Three measures of biological interaction were used to investigate biological interaction between physical inactivity and low HDL-C, as described by Andersson and colleagues [25]: the relative excess risk due to interaction (RERI); the attributable proportion due to interaction (AP); and the synergy index (S) (RERI and AP would be equal to 0 and S would be equal to 1 if there were no biological interaction [25]). Population attributable risks were calculated based on all-cause mortality. All analyses were performed using SPSS version 22 (IBM Inc.).

## Results

There were 37,059 participants included in the present study. Some 90% of participants were white and other broad ethnic groups included black (2.6%), Asian (6.7%), and Chinese (1.9%). Table 1 shows participants’ characteristics at baseline according to the meeting of physical activity guidelines and HDL-C concentration. Some 30% of participants met physical activity guidelines. In those who met physical activity guidelines, age tended to be lower, the proportion of men tended to be higher, the proportion reporting longstanding illness tended to be lower, and the proportion employed in professional occupations tended to be higher. Cardiovascular disease prevalence and CVD medication use were lower in those who met physical activity guidelines than those who did not. Body mass index, total cholesterol, and systolic blood pressure tended to be lower in those who met physical activity guidelines, but there was considerable overlap with those who did not meet physical activity guidelines. High-density lipoprotein cholesterol concentration was around 1.6 mmol L^−1^ in those with normal concentrations and around 1.0 mmol L^−1^ in those with low concentrations. Low HDL-C was identified in 15% of those who met physical activity guidelines and 22% of those who did not. The partial correlation adjusted for age and sex between non-occupational physical activity (MET-h wk^−1^) and HDL-C (mmol L^−1^) was 0.10 (*p* < 0.001). Table S1 in the Online Supplement shows participants’ age, HDL-C and physical activity by survey and survey year.

**Table 1:** Participants’ characteristics at baseline (n = 37,059)

	Meeting physical activity guidelines (n = 10,898)^a^		Not meeting physical activity guidelines (n = 26,161)^a^	
	Normal HDL-C^b^	Low HDL-C^b^	Normal HDL-C^b^	Low HDL-C^b^
Age (years)	41 ± 15	38 ± 14	50 ± 16	47 ± 16
Sex (% male)	51.9	53.8	42.2	42.5
Body mass index (kg m^−2^)	25.5 ± 3.9	27.8 ± 4.8	26.5 ± 4.6	28.8 ± 5.2
HDL-C (mmol L^−1^)	1.6 ± 0.4	1.0 ± 0.2	1.6 ± 0.4	1.0 ± 0.2
Total cholesterol (mmol L^−1^)	5.4 ± 1.1	5.1 ± 1.1	5.7 ± 1.1	5.5 ± 1.2
Systolic blood pressure (mm Hg)	126.2 ± 15.8	126.6 ± 15.1	131.0 ± 19.1	131.1 ± 19.1
Smoker (%)	17.6	27.0	23.5	31.9
Non-occupational activity (MET-h wk^−1^)	49.6 ± 46.2	49.0 ± 57.6	16.8 ± 25.3	15.0 ± 24.0
Longstanding illness (%)	30.6	35.2	44.6	49.5
Cardiovascular disease prevalence (%)^c^	1.1	1.5	4.1	6.7
Cardiovascular disease medication (%)^d^	13.0	16.6	25.2	29.6
Social occupational group (% professional)	7.7	5.8	4.4	3.3

^a^Here, physical activity excludes domestic activity and meeting physical activity guidelines is defined as taking part in at least 150 min per week of moderate-intensity physical activity, or at least 75 min per week of vigorous-intensity physical activity, or any combination of moderate- and vigorous-intensity physical activity equivalent to at least 7.5 MET-h wk^−1^

^b^Here, low HDL-C defined as <1.03 mmol L^−1^ in men and <1.30 mmol L^−1^ in women

^c^Cardiovascular disease prevalence includes doctor-diagnosed heart attack, stroke, or angina

^d^Cardiovascular disease medication includes beta blockers, ACE inhibitors, diuretics, calcium blockers, and lipid lowering agents

Data are mean ± SD unless indicated otherwise

There were 2250 deaths from all causes, including 649 from CVD, during 326,016 person years of follow-up (mean: 8.8 years of follow-up [range: 2–15 years]). Survival curves for all-cause mortality and CVD mortality according to exposure are shown in the Online Supplement (Figures S1 and S2, respectively). Table 2 shows the associations between physical activity, HDL-C and all-cause mortality with low HDL-C defined as <1.03 mmol L^−1^ in men and <1.30 mmol L^−1^ in women. Compared with those who met physical activity guidelines and whose HDL-C concentration was normal (the reference group), the fully-adjusted hazard ratio for all-cause mortality was 1.07 and the 95% confidence interval included zero (0.75, 1.53) in those who met physical activity guidelines and whose HDL-C concentration was low. Compared with the reference group, the fully-adjusted hazard ratio for all-cause mortality was 1.37 (95% CI: 1.16, 1.61) in those who did not meet physical activity guidelines and whose HDL-C concentration was normal, and 1.65 (95% CI: 1.37, 1.98) in those who did not meet physical activity guidelines and whose HDL-C concentration was low. The associations were similar in magnitude and direction in the subsamples with medication data (Table S2, Online Supplement) and aged 40 years and older (Table S3, Online Supplement). The associations between physical activity, HDL-C and all-cause mortality were also similar after further adjustment for survey year (Table S4, Online Supplement). The associations between the covariates and mortality are shown in Table S5 in the Online Supplement.

**Table 2:** Cox proportional hazard ratios (HR) for associations between physical activity, high-density lipoprotein cholesterol (HDL-C) and all-cause and cardiovascular disease mortality with low HDL-C defined differently in men and women (n = 37,059)

Meeting physical activity guidelines^a^	HDL-C^b^	Deaths/N	Age- and sex-adjusted HR (95% CI)	Fully-adjusted HR (95% CI)^c^
All-cause mortality				
Yes	Normal	170/9234	1.0 (Reference)	1.0 (Reference)
Yes	Low	40/1664	1.13 (0.80, 1.60)	1.07 (0.75, 1.53)
No	Normal	1514/20,487	1.58 (1.35, 1.86)	1.37 (1.16, 1.61)
No	Low	526/5674	1.98 (1.66, 2.35)	1.65 (1.37, 1.98)
Cardiovascular disease mortality				
Yes	Normal	48/9234	1.0 (Reference)	1.0 (Reference)
Yes	Low	7/1664	0.71 (0.32, 1.56)	0.60 (0.25, 1.40)
No	Normal	418/20,487	1.36 (1.00, 1.84)	1.11 (0.82, 1.52)
No	Low	176/5674	2.13 (1.54, 2.94)	1.63 (1.16, 2.27)

^a^Here, physical activity excludes domestic activity and meeting physical activity guidelines is defined as taking part in at least 150 min per week of moderate-intensity physical activity, or at least 75 min per week of vigorous-intensity physical activity, or any combination of moderate- and vigorous-intensity physical activity equivalent to at least 7.5 MET-h wk^−1^

^b^Here, low HDL-C defined as <1.03 mmol L^−1^ in men and <1.30 mmol L^−1^ in women

^c^Model adjusted for age, sex, smoking, total cholesterol, systolic blood pressure, body mass index, longstanding illness, and social class

**Fig. 1:**
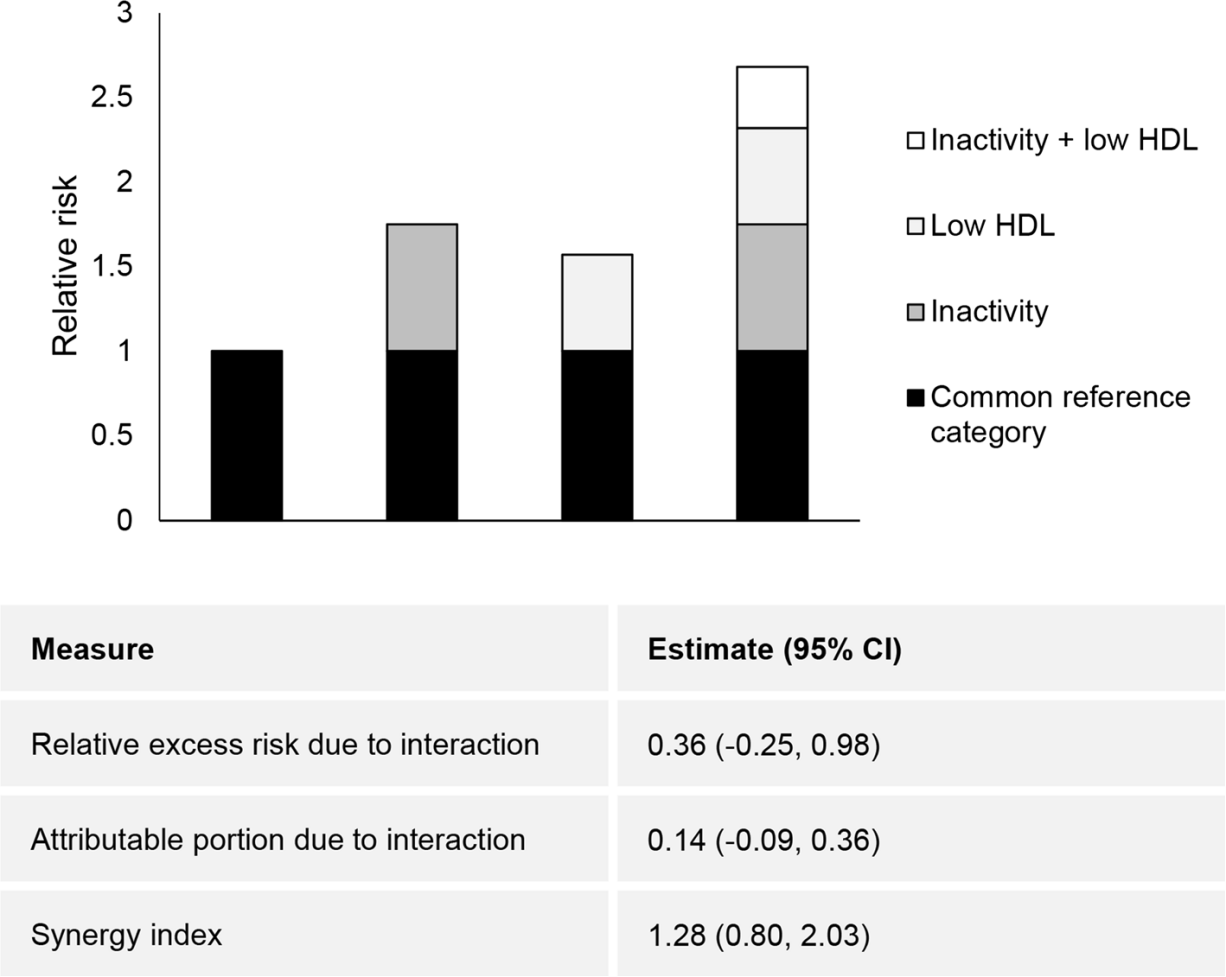
Relative risk of all-cause mortality with contributions from different exposure categories. The relative excess risk due to interaction and the attributable proportion due to interaction are equal to 0 and the synergy index is equal to 1 when there is no biological interaction [25]

Table 2 also shows the associations between physical activity, HDL-C and CVD mortality with low HDL-C defined as <1.03 mmol L^−1^ in men and <1.30 mmol L^−1^ in women. Compared with those who met physical activity guidelines and whose HDL-C concentration was normal (the reference group), the fully-adjusted hazard ratio for CVD mortality was 0.60 (95% CI: 0.25, 1.40) in those who met physical activity guidelines and whose HDL-C concentration was low. Compared with the reference group, the fully-adjusted hazard ratio for CVD mortality was 1.11 (95% CI: 0.82, 1.52) in those who did not meet physical activity guidelines and whose HDL-C concentration was normal, and 1.63 (95% CI: 1.16, 2.27) in those who did not meet physical activity guidelines and whose HDL-C concentration was low. Table 3 shows the associations between physical activity and mortality with low HDL-C defined as <1.03 mmol L^−1^ in men and women. The use of a single definition of low HDL-C and the resultant reallocation of women had little impact on the magnitudes of the hazard ratios or the widths of the confidence intervals. Figure 1 shows the relative risk of all-cause mortality with contributions from physical inactivity and low HDL-C. There was no statistically significant evidence of biological interaction between physical inactivity and low HDL-C.

**Table 3:** Cox proportional hazard ratios (HR) for associations between physical activity, high-density lipoprotein cholesterol (HDL-C) and all-cause and cardiovascular disease mortality with low HDL-C defined similarly in men and women (n = 37,059)

Meeting physical activity guidelines^a^	HDL-C^b^	Deaths/N	Age- and sex-adjusted HR (95% CI)	Fully-adjusted HR (95% CI)^c^
All-cause mortality				
Yes	Normal	180/9774	1.0 (Reference)	1.0 (Reference)
Yes	Low	30/1124	1.13 (0.74, 1.66)	1.05 (0.70, 1.56)
No	Normal	1679/22,682	1.60 (1.37, 1.87)	1.38 (1.17, 1.61)
No	Low	361/3479	1.96 (1.64, 2.35)	1.65 (1.37, 1.99)
Cardiovascular disease mortality				
Yes	Normal	48/9774	1.0 (Reference)	1.0 (Reference)
Yes	Low	7/1124	0.96 (0.44, 2.13)	0.79 (0.34, 1.86)
No	Normal	472/22,682	1.49 (1.10, 2.01)	1.19 (0.88, 1.63)
No	Low	122/3479	2.20 (1.57, 3.08)	1.72 (1.20, 2.43)

^a^Here, physical activity excludes domestic activity and meeting physical activity guidelines is defined as taking part in at least 150 min per week of moderate-intensity physical activity, or at least 75 min per week of vigorous-intensity physical activity, or any combination of moderate- and vigorous-intensity physical activity equivalent to at least 7.5 MET-h wk^−1^

^b^Here, low HDL-C defined as <1.03 mmol L^−1^ in men and women

^c^Model adjusted for age, sex, smoking, total cholesterol, systolic blood pressure, body mass index, longstanding illness, and social class

Table S6 in the Online Supplement shows the calculation of population attributable risks based on all-cause mortality. It was calculated that, if everybody were to adhere to physical activity guidelines, there would be a reduction of 4.1 new mortality cases per 100 population. Such a reduction represents a 68.2% reduction of the incidence in the population. It was also calculated that, if everybody were to have normal HDL-C, there would be a reduction of 0.4 new mortality cases per 100 population. Such a reduction represents a 6.6% reduction of the incidence in the population.

## Discussion

The objective of this study was to investigate associations between leisure-time physical activity, low HDL-C and mortality. The main findings were that the risks of all-cause and CVD mortality were similar in both those who met physical activity guidelines and whose HDL-C concentration was normal and those who met physical activity guidelines and whose HDL-C concentration was low. There was no statistically significant evidence of biological interaction between physical inactivity and low HDL-C; rather, the relative risks of all-cause mortality were additive. This study supports the notion that physical activity be recommended in those with low HDL-C.

According to ATP III, therapeutic lifestyle changes include: reduced intake of saturated fats and cholesterol; therapeutic dietary options to enhance low-density lipoprotein cholesterol lowering (plant stanols/sterols and increased viscous fibre); weight control, and increased physical activity [1]. Therapeutic lifestyle changes and drugs are explicitly recommended in those with high low-density lipoprotein cholesterol concentrations according to CVD risk [1, 2]. The roles of therapeutic lifestyle changes and drugs are ambiguous in those with low HDL-C concentrations [1, 2]. Around 20% of adults in the present study had low HDL-C and 37% of 15,252 white men and women in four American studies had low HDL-C [26]. Data from the four American studies suggest that coronary heart disease incidence and all-cause mortality rates are higher in adults with low HDL-C than high HDL-C (<1.04 and ≥50 mmol L^−1^, respectively) [26]. Around 15% of physically active participants in the present study had low HDL-C. Physical inactivity is regarded as a cause of low HDL-C levels [1] and exercise training tends to increase HDL-C [3]; however, those with low HDL-C or ‘isolated low HDL-C’ may be resistant to the HDL-raising effect of exercise (isolated low HDL-C refers to low HDL-C in the absence of other lipid abnormalities) [9–12]. The fibrate class of drugs was tentatively recommended in those with low HDL-C or high triglycerides/low HDL-C [2]. While fibrate class can be effective in the secondary prevention of non-fatal stroke, non-fatal myocardial infarction, and vascular death, the beneficial effect relies on the inclusion of clofibrate data [8]. Clofibrate was discontinued in 2002 because of unacceptably large adverse effects. The present study suggests that physical activity could be recommended in those with low HDL-C because it is associated with reduced risk of mortality. The present study also suggests that the relationships between physical activity, low HDL-C and mortality are similar whether low HDL-C is defined using the single or sex-specific definitions of ATP III [1]. Sex-specific definitions of low HDL-C are recommended in ATP III [1]; however, the present study and a meta-analysis of 23 studies [7] suggest that the use of a single definition or sex-specific definitions has little influence on the association with CVD (the individual participant data meta-analysis included 191,452 Asians and 28,608 non-Asians from the Asia–Pacific region [7]).

The inverse associations between physical activity and mortality we have observed in those with normal and low HDL-C are biologically plausible. It is well documented in the general population that exercise training increases cardiorespiratory fitness, increases insulin sensitivity, reduces markers of inflammation, reduces blood pressure, improves vascular function, and improves lipids and lipoproteins [27]. The evidence in those with low HDL-C is limited, but it would seem that exercise training also increases cardiorespiratory fitness [9–12], reduces triglyceride concentration [10], and improves the chemical and physical characteristics of low-density lipoprotein cholesterol [9]. The effect of exercise training on cardiorespiratory fitness may be particularly important because fitness may be a stronger predictor of mortality than smoking, high cholesterol, and a number of traditional risk factors [28]. It has also been estimated that, if physical inactivity were to decrease by 25%, some 9% of deaths could be avoided (ranging from 4% in low-income countries to 11% in high-income countries) [29]. The population attributable risk was higher in the present study because we estimated what would happen if everybody were to adhere to physical activity guidelines. High-density lipoprotein cholesterol efflux capacity is inversely associated with cardiovascular events [30] and it is also plausible that exercise improves HDL-C functionality without modifying HDL-C concentration [31].

There are few head-to-head comparisons of the effects of exercise and drug interventions on mortality [32]. Network meta-analysis can be used to combine direct evidence (from trials that include a head-to-head comparison) and indirect evidence (from a network of trials that do not include the comparison). Naci and Ioannidis [32] reviewed the literature to May 2013 and found 34 exercise interventions (10,984 participants), 165 drug trials (161,787 participants), and no head-to-head comparisons in the secondary prevention of coronary heart disease. Network meta-analysis revealed no statistically detectable differences in mortality between the exercise interventions and drug trials [32]. Naci and Ioannidis [32] suggested that there was a preference for drug trials in medical research and that this preference had influenced clinical practice guidelines; for example, they said, statins were only recommended after lifestyle interventions had been exhausted in earlier versions of the National Cholesterol Education Program guidelines [33].

Our study has some limitations. Ninety per cent of participants were white and the results may not be generalizable to other groups. Diet was not assessed. Physical activity and HDL-C were only assessed at baseline and we cannot account for changes over time. Physical activity was self-reported; however, questionnaires are still regarded as the mainstay of established surveillance studies such as HSE and SHS [34]. We were unable to distinguish between low HDL-C and isolated low HDL-C because triglyceride concentrations were not available; however, there is little distinction between the causes and treatment of both conditions [1, 2]. We cannot discount the possibility of reverse causation; however, in keeping with previous analyses in the same cohorts [35, 36], we excluded deaths in the first 24 months of follow-up and we adjusted for longstanding illness. The number of CVD deaths was relatively low, which resulted in low statistical power in some comparisons.

In conclusion, the present study suggests that the risks of all-cause and CVD mortality are similar in both those who meet physical activity guidelines and whose HDL-C concentration is normal and those who meet physical activity guidelines and whose HDL-C concentration is low. This novel finding supports the notion that leisure-time physical activity be recommended in those with low HDL-C concentration.

## Electronic supplementary material

Below is the link to the electronic supplementary material.

Supplementary material 1 (DOCX 61 kb)

## References

[1] National cholesterol education program. Third report of the national cholesterol education program (NCEP) expert panel on detection, evaluation, and treatment of high blood cholesterol in adults (adult treatment panel III) final report. Circulation. 2002;106(25):3143–421. 12485966

[2] Grundy SM, Cleeman JI, Merz CN (2004). Implications of recent clinical trials for the national cholesterol education program adult treatment panel III guidelines. Circulation.

[3] Kodama S, Tanaka S, Saito K (2007). Effect of aerobic exercise training on serum levels of high-density lipoprotein cholesterol: a meta-analysis. Arch Intern Med.

[4] Arem H, Moore SC, Patel A (2015). Leisure time physical activity and mortality: a detailed pooled analysis of the dose-response relationship. JAMA Intern Med.

[5] O’Donovan G, Lee IM, Hamer M, Stamatakis E. Association of “Weekend Warrior”and other leisure time physical activity patterns with risks for all-cause, cardiovascular disease, and cancer mortality. JAMA Intern Med. 10.1001/jamainternmed.2016.8014. 10.1001/jamainternmed.2016.8014 28097313

[6] Goldbourt U, Yaari S, Medalie JH (1997). Isolated low HDL cholesterol as a risk factor for coronary heart disease mortality. A 21-year follow-up of 8000 men. Arterioscler Thromb Vasc Biol.

[7] Huxley RR, Barzi F, Lam TH (2011). Isolated low levels of high-density lipoprotein cholesterol are associated with an increased risk of coronary heart disease: an individual participant data meta-analysis of 23 studies in the Asia-Pacific region. Circulation.

[8] Wang D, Liu B, Tao W, Hao Z, Liu M. Fibrates for secondary prevention of cardiovascular disease and stroke. Cochrane Database Syst Rev 2015;10:CD009580. 10.1002/14651858.CD009580.pub2 PMC6494578 26497361

[9] Houmard JA, Bruno NJ, Bruner RK, McCammon MR, Israel RG, Barakat HA (1994). Effects of exercise training on the chemical composition of plasma LDL. Arterioscler Thromb.

[10] Nicklas BJ, Katzel LI, Busby-Whitehead J, Goldberg AP (1997). Increases in high-density lipoprotein cholesterol with endurance exercise training are blunted in obese compared with lean men. Metabolism.

[11] Zmuda JM, Yurgalevitch SM, Flynn MM (1998). Exercise training has little effect on HDL levels and metabolism in men with initially low HDL cholesterol. Atherosclerosis.

[12] Couillard C, Despres JP, Lamarche B (2001). Effects of endurance exercise training on plasma HDL cholesterol levels depend on levels of triglycerides: evidence from men of the health, risk factors, exercise training and genetics (HERITAGE) family study. Arterioscler Thromb Vasc Biol.

[13] Craig R, Deverill C, Pickering K, Prescott A. methodology and response. In: Bromley C, Sproston K, Shelton N, editors. The scottish health survey 2003. volume 4: technical report. Edinburgh: Crown; 2005. p. 1–48.

[14] Craig R, Mindell J, Hirani V, editors. Health survey for England 2008. Volume 2: methods and documentation. London: National centre for social research; 2010.

[15] Stamatakis E, Hillsdon M, Primatesta P (2007). Domestic physical activity in relationship to multiple CVD risk factors. Am J Prev Med.

[16] Scholes S, Coombs N, Pedisic Z (2014). Age- and sex-specific criterion validity of the health survey for England physical activity and sedentary behavior assessment questionnaire as compared with accelerometry. Am J Epidemiol.

[17] Joint health surveys unit. Health survey for England physical activity validation study: substantive report. Leeds: information centre for health and social care. 2007.

[18] Ainsworth BE, Haskell WL, Herrmann SD (2011). 2011 compendium of physical activities: a second update of codes and MET values. Med Sci Sports Exerc.

[19] World health organisation. Global recommendations on physical activity for health. Secondary global recommendations on physical activity for health. 2010. http://www.who.int/dietphysicalactivity/factsheet_recommendations/en/.

[20] Sabia S, Dugravot A, Kivimaki M, Brunner E, Shipley MJ, Singh-Manoux A (2012). Effect of intensity and type of physical activity on mortality: results from the Whitehall II cohort study. Am J Public Health.

[21] Stamatakis E, Hamer M, Lawlor DA (2009). Physical activity, mortality, and cardiovascular disease: is domestic physical activity beneficial? The Scottish Health Survey–1995, 1998, and 2003. Am J Epidemiol.

[22] Craig R, Deverill C, Pickering K, Spronston K, Mindell J (2006). Quality control of blood, saliva and urine analytes. Methodology and documentation.

[23] Collins GS, Mallett S, Omar O, Yu LM (2011). Developing risk prediction models for type 2 diabetes: a systematic review of methodology and reporting. BMC Med.

[24] Thompson PD, Franklin BA, Balady GJ (2007). Exercise and acute cardiovascular events placing the risks into perspective: a scientific statement from the American heart association council on nutrition, physical activity, and metabolism and the council on clinical cardiology. Circulation.

[25] Andersson T, Alfredsson L, Kallberg H, Zdravkovic S, Ahlbom A (2005). Calculating measures of biological interaction. Eur J Epidemiol.

[26] Gordon DJ, Probstfield JL, Garrison RJ (1989). High-density lipoprotein cholesterol and cardiovascular disease. Four prospective American studies. Circulation.

[27] Hamer M, O’Donovan G (2010). Cardiorespiratory fitness and metabolic risk factors in obesity. Curr Opin Lipidol.

[28] Ross R, Blair SN, Arena R (2016). Importance of assessing cardiorespiratory fitness in clinical practice: a case for fitness as a clinical vital sign: a scientific statement from the American heart association. Circulation.

[29] Lee IM, Shiroma EJ, Lobelo F, Puska P, Blair SN, Katzmarzyk PT (2012). Effect of physical inactivity on major non-communicable diseases worldwide: an analysis of burden of disease and life expectancy. Lancet.

[30] Rohatgi A, Khera A, Berry JD (2014). HDL cholesterol efflux capacity and incident cardiovascular events. N Engl J Med.

[31] Trejo-Gutierrez JF, Fletcher G (2007). Impact of exercise on blood lipids and lipoproteins. J Clin Lipidol.

[32] Naci H, Ioannidis JP (2015). Comparative effectiveness of exercise and drug interventions on mortality outcomes: metaepidemiological study. Br J Sports Med.

[33] Program National Cholesterol Education (1993). Summary of the second report of the national cholesterol education program (NCEP) expert panel on detection, evaluation, and treatment of high blood cholesterol in adults (adult treatment panel II). JAMA.

[34] Pedisic Z, Bauman A (2015). Accelerometer-based measures in physical activity surveillance: current practices and issues. Br J Sports Med.

[35] O’Donovan G, Lee IM, Hamer M, Stamatakis E (2017). Association of “Weekend Warrior” and other leisure time physical activity patterns with risks for all-cause, cardiovascular disease, and cancer mortality. JAMA Internal Med.

[36] Hamer M, O’Donovan G, Stensel D, Stamatakis E (2017). Normal-weight central obesity and risk for mortality. Ann Intern Med.

