# Supplementary material for: The association between leisure-time physical activity, low HDL-cholesterol and mortality in a pooled analysis of nine population-based cohorts
Source: Eur J Epidemiol. 2017 Jun 30;32(7):559–66. doi: 10.1007/s10654-017-0280-9 (PMC5570782; doi:10.1007/s10654-017-0280-9)
Supplement: Supplementary file 1 — Supplementary material 1 (DOCX 61 kb) [file 10654_2017_280_MOESM1_ESM.docx]

| 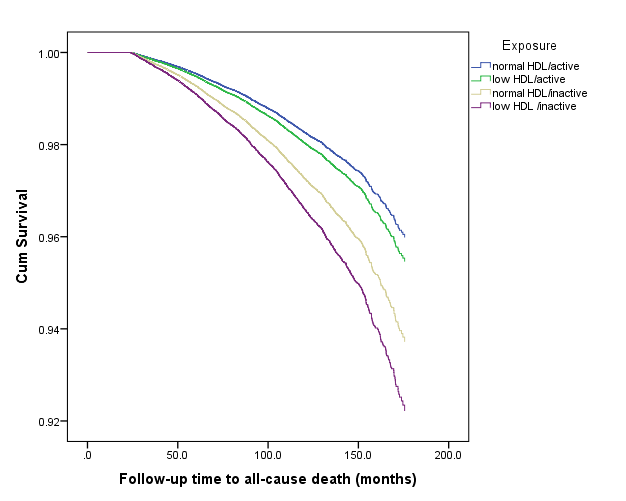 |
| --- |
| **Figure S1.** Survival curves for all-cause mortality according to exposure: meeting physical activity guidelines and normal HDL-C concentration (reference group); meeting physical activity guidelines and low HDL-C concentration; not meeting physical activity guidelines and normal HDL-C concentration; not meeting physical activity guidelines and low HDL-C concentration. Here, low HDL-C defined as <1.03 mmol·L^-1^ in men and <1.30 mmol·L^-1^ in women. |

| 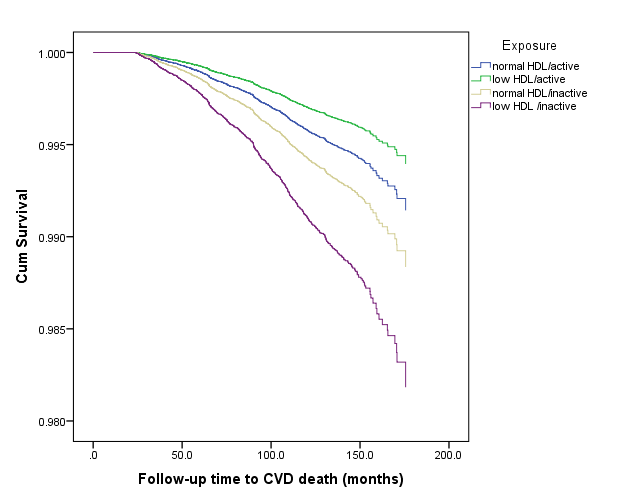 |
| --- |
| **Figure S2.** Survival curves for cardiovascular disease (CVD) mortality according to exposure: meeting physical activity guidelines and normal HDL-C concentration (reference group); meeting physical activity guidelines and low HDL-C concentration; not meeting physical activity guidelines and normal HDL-C concentration; not meeting physical activity guidelines and low HDL-C concentration. Here, low HDL-C defined as <1.03 mmol·L^-1^ in men and <1.30 mmol·L^-1^ in women. |

**Table S1.** Participants’ age, high-density lipoprotein cholesterol (HDL-C), and physical activity by survey and survey year*

| Survey and year | N | Age, years | HDL-C,  mmol·L^-1^ | Physical activity, min·wk^-1^ |
| --- | --- | --- | --- | --- |
| HSE1998 | 8265 | 47.3±17.1 | 1.43±0.41 | 117±254 |
| HSE1999 | 2637 | 42.5±16.2 | 1.39±0.42 | 76±327 |
| HSE2003 | 5524 | 48.2±16.5 | 1.54±0.39 | 83±203 |
| HSE2004 | 1336 | 41.6±14.5 | 1.42±0.36 | 77±186 |
| HSE2006 | 4745 | 48.0±16.1 | 1.52±0.39 | 158±321 |
| HSE2008 | 4274 | 47.8±16.4 | 1.52±0.38 | 177±315 |
| SHS1995 | 4168 | 41.9±12.9 | 1.43±0.44 | 79±180 |
| SHS1998 | 4019 | 46.8±15.3 | 1.44±0.44 | 122±276 |
| SHS2003 | 2088 | 50.7±16.4 | 1.52±0.37 | 153±306 |
| Overall | 37059 | 46.6±16.2 | 1.47±0.41 | 118±269 |

*Values are mean±standard deviation. Physical activity includes moderate- and vigorous-intensity physical activities.

**Table S2.** Cox proportional hazard ratios (HR) for associations between physical activity, high-density lipoprotein cholesterol (HDL-C) and all-cause mortality in subsample with medication data (n=18,928)

| Meeting physical activity guidelines* | HDL-C† | Deaths/N | Age- and sex-adjusted HR (95% CI) | Fully-adjusted HR (95% CI)‡ |
| --- | --- | --- | --- | --- |
| Yes | Normal | 104/3653 | 1.0 (Reference) | 1.0 (Reference) |
| Yes | Low | 22/744 | 0.84 (0.53, 1.33) | 0.79 (0.49, 1.26) |
| No | Normal | 1181/11,116 | 1.63 (1.33, 2.00) | 1.39 (1.13, 1.70) |
| No | Low | 442/3,415 | 1.95 (1.57, 2.41) | 1.61 (1.29, 2.00) |

*Here, physical activity excludes domestic activity and meeting physical activity guidelines is defined as taking part in at least 150 minutes per week of moderate-intensity physical activity, or at least 75 minutes per week of vigorous-intensity physical activity, or any combination of moderate- and vigorous-intensity physical activity equivalent to at least 7.5 MET-hr·wk^-1^.

† Here, low HDL-C defined as <1.03 mmol·L^-1^ in men and <1.30 mmol·L^-1^ in women.

‡Model adjusted for age, sex, smoking, total cholesterol, systolic blood pressure, body mass index, longstanding illness, and social class.

**Table S3.** Cox proportional hazard ratios (HR) for associations between physical activity, high-density lipoprotein cholesterol (HDL-C) and all-cause mortality in subsample aged 40 years and older (n=23,386)

| Meeting physical activity guidelines* | HDL-C† | Deaths/N | Age- and sex-adjusted HR (95% CI) | Fully-adjusted HR (95% CI)‡ |
| --- | --- | --- | --- | --- |
| Yes | Normal | 143/4714 | 1.0 (Reference) | 1.0 (Reference) |
| Yes | Low | 34/694 | 1.16 (0.80, 1.67) | 1.12 (0.77, 1.64) |
| No | Normal | 1462/14325 | 1.67 (1.40, 1.98) | 1.44 (1.20, 1.71) |
| No | Low | 512/3653 | 2.12 (1.76, 2.55) | 1.78 (1.46, 2.15) |

*Here, physical activity excludes domestic activity and meeting physical activity guidelines is defined as taking part in at least 150 minutes per week of moderate-intensity physical activity, or at least 75 minutes per week of vigorous-intensity physical activity, or any combination of moderate- and vigorous-intensity physical activity equivalent to at least 7.5 MET-hr·wk^-1^.

† Here, low HDL-C defined as <1.03 mmol·L^-1^ in men and <1.30 mmol·L^-1^ in women.

‡Model adjusted for age, sex, smoking, total cholesterol, systolic blood pressure, body mass index, longstanding illness, and social class.

**Table S4.** Cox proportional hazard ratios (HR) for associations between physical activity, high-density lipoprotein cholesterol (HDL-C) and all-cause mortality with adjustment for survey year and other potential confounders

| Meeting physical activity guidelines* | HDL-C† | Deaths/N | Fully-adjusted HR (95% CI)‡ |
| --- | --- | --- | --- |
| Yes | Normal | 170/9,234 | 1.0 (Reference) |
| Yes | Low | 40/1,664 | 1.03 (0.73, 1.47) |
| No | Normal | 1514/20,487 | 1.38 (1.17, 1.63) |
| No | Low | 526/5,674 | 1.64 (1.37, 1.97) |

*Here, physical activity excludes domestic activity and meeting physical activity guidelines is defined as taking part in at least 150 minutes per week of moderate-intensity physical activity, or at least 75 minutes per week of vigorous-intensity physical activity, or any combination of moderate- and vigorous-intensity physical activity equivalent to at least 7.5 MET-hr·wk^-1^.

† Here, low HDL-C defined as <1.03 mmol·L^-1^ in men and <1.30 mmol·L^-1^ in women.

‡Model adjusted for age, sex, smoking, total cholesterol, systolic blood pressure, body mass index, longstanding illness, social class, and survey year.

**Table S5.** Associations between the covariates and mortality

| Variable | | Hazard ratio (95% CI)* |
| --- | --- | --- |
| Age (per year) | | 1.10 (1.09, 1.11) |
| Male sex | | 1.46 (1.32, 1.62) |
| Smoking habit | |  |
|  | Never | 1.0 (Reference) |
|  | Ex-smoker | 1.32 (1.19, 1.46) |
|  | <10 per day | 1.91 (1.58, 2.31) |
|  | 10-19 per day | 2.32 (1.99, 2.69) |
|  | 20+ per day | 3.45 (2.98, 3.99) |
| Social occupational group | |  |
|  | Professional | 1.0 (Reference) |
|  | Managerial | 1.29 (0.98, 1.69) |
|  | Skilled non-manual | 1.41 (1.07, 1.86) |
|  | Skilled manual | 1.49 (1.14, 1.95) |
|  | Semi-skilled manual | 1.42 (1.08, 1.87) |
|  | Unskilled manual | 1.62 (1.21, 2.17) |
|  | Other | 2.26 (1.24, 4.13) |
| Meeting physical activity guidelines | | 0.71 (0.61, 0.83) |
| Total cholesterol† | | 0.99 (0.94, 1.03) |
| HDL-C† | | 0.97 (0.93, 1.01) |
| Systolic blood pressure† | | 1.08 (1.04, 1.12) |
| Body mass index | |  |
|  | Underweight | 2.59 (1.87, 3.59) |
|  | Normal weight | 1.0 (Reference) |
|  | Overweight | 0.75 (0.68, 0.83) |
|  | Obese | 0.83 (0.74, 0.94) |
| Long-term illness | | 1.40 (1.28, 1.53) |

*Hazard ratios are mutually adjusted for all variables present.

†Effects estimated per standard deviation.

**Table S6.** Calculation of population attributable risks based on all-cause mortality

*A. Population attributable risk for physical activity*

| Meets physical activity guidelines | Cases  (all cause mortality) | Non-cases | *Total* |
| --- | --- | --- | --- |
| No | 2040 | 24121 | 26161 |
| Yes | 210 | 10688 | 10898 |
| *Total* | 2250 | 34809 | 37059 |

Incidence in the exposed (non-active) (I_e_ ) = 7.8 per 100

Incidence in the unexposed (active) (I_u_ ) = 1.9 per 100

Incidence in both combined (I_p_ ) = 6.1 per 100.

Attributable risk (I_e_ – I_u_) = 5.87 (75.3%)

Population attributable risk (I_p_ – I_u_) = 4.14 (68.2%).

**A reduction of 4.1 new mortality cases per 100 population (exposed and unexposed) is expected if everybody adheres to the physical activity guideline. Such reduction represents a 68.2% reduction of the incidence in the population.**

*B. Population attributable risk for HDL-C*

| HDL-C cut point | Cases  (all cause mortality) | Non-cases | Total |
| --- | --- | --- | --- |
| Low | 566 | 6772 | 7338 |
| Normal | 1684 | 28037 | 29721 |
| *Total* | 2250 | 34809 | 37059 |

Incidence in the exposed (low HDL) (I_e_ ) = 7.7 per 100

Incidence in the unexposed (normal HDL) (I_u_ ) = 5.7 per 100

Incidence in both combined (I_p_ ) = 6.1 per 100.

Attributable risk (I_e_ – I_u_) = 2.0 (26.0%)

Population attributable risk (I_p_ – I_u_) = 0.4 (6.6%).

**A reduction of 0.4 new mortality cases per 100 population (exposed and unexposed) is expected if everybody has normal HDL-C. Such reduction represents a 6.6% reduction of the incidence in the population.**
